# Video-calls to reduce loneliness and social isolation within care environments for older people: an implementation study using collaborative action research

**DOI:** 10.1186/s12877-018-0746-y

**Published:** 2018-03-02

**Authors:** Sonam Zamir, Catherine Hagan Hennessy, Adrian H Taylor, Ray B Jones

**Affiliations:** 1 Drake Circus, School of Nursing and Midwifery, University of Plymouth, Plymouth, Devon PL4 8AA England; 2 Faculty of Social Sciences, University of Stirling, Stirling, FK9 4LA Scotland; 3 University of Plymouth Peninsula Schools of Medicine & Dentistry, ITTC Building, Tamar Science Park, Derriford, Plymouth, Devon PL6 8BX England; 4 Drake Circus, School of Nursing and Midwifery, University of Plymouth, Plymouth, Devon PL4 8AA England

**Keywords:** Skype, Video-calls, Intervention, Collaborative, Action, Research, Elderly loneliness, Isolation, Care-settings

## Abstract

**Background:**

Older people in care may be lonely with insufficient contact if families are unable to visit. Face-to-face contact through video-calls may help reduce loneliness, but little is known about the processes of engaging people in care environments in using video-calls. We aimed to identify the barriers to and facilitators of implementing video-calls for older people in care environments.

**Methods:**

A collaborative action research (CAR) approach was taken to implement a video-call intervention in care environments. We undertook five steps of recruitment, planning, implementation, reflection and re-evaluation, in seven care homes and one hospital in the UK. The video-call intervention ‘Skype on Wheels’ (SoW) comprised a wheeled device that could hold an iPad and handset, and used Skype to provide a free video-call service. Care staff were collaborators who implemented the intervention within the care-setting by agreeing the intervention, recruiting older people and their family, and setting up video-calls. Field notes and reflective diaries on observations and conversations with staff, older people and family were maintained over 15 months, and analysed using thematic analysis.

**Results:**

Four care homes implemented the intervention. Eight older people with their respective social contacts made use of video-calls. Older people were able to use SoW with assistance from staff, and enjoyed the use of video-calls to stay better connected with family. However five barriers towards implementation included staff turnover, risk averseness, the SoW design, lack of family commitment and staff attitudes regarding technology.

**Conclusions:**

The SoW intervention, or something similar, could aid older people to stay better connected with their families in care environments, but if implemented as part of a rigorous evaluation, then co-production of the intervention at each recruitment site may be needed to overcome barriers and maximise engagement.

## Background

Loneliness and social isolation among older people may be detrimental to well-being [1], quality of life [2] and cognitive decline [3]. Technological interventions have been developed that may reduce loneliness for dementia patients through telephone ‘be-friending’ projects [4, 5], and the use of the internet [6, 7]. Even so, social media and emailing provide less personal connectivity than face-to-face contact with a loved one, and may even add to the feeling of loneliness and isolation. [8]. Previous studies have revealed that face-to-face contact through video-calls may be more useful for older people than telephone calls or written correspondence in reducing loneliness [9–11].Technologies such as iPads are easily mobile and can be used for video-calls using software such as Skype, a free tele-service. Older people may be capable of using iPads and Skype, but not all care environments provide this technology [12]. There is therefore a need to better understand the factors influencing the use of technology to reduce loneliness and isolation, and how it may be useful for older people.

Loneliness and social isolation have been defined in various ways. Researchers now believe that loneliness is a perceptual concept whereas social isolation is defined as the lack of ‘structural’ and ‘functional’ social support [13]. Structural social support is normally assessed by the size of one’s social networks and frequency of contacts within that network. On the other hand, functional social support is a subjective judgment of the quality or perceived value of emotional and informational support, provided by those within their social network [14].

In terms of the quality and perceived value of support, Porges’s social engagement and attachment theory posits the importance of seeing one another’s faces during communication [15]. This is because body language influences both the expression and receptivity of social cues, consequently reducing perceived social distance. In particular, use of facial expressions, eye gaze, and head orientation is important for social engagement, which can be lost in asynchronous communication and telephone calls. These expressions can be seen as an active social engagement system reducing psychological distance, and can influence perception in the engagement of others [15]. Porges’s theory places importance on the role of face-to-face interaction in maintaining social bonds, and thus reducing feelings of loneliness and social isolation.

In modern society, face-to-face communication with family members has declined creating a need to find alternative methods to maintain communication. Socialisation interventions that incorporate face-to-face communication through video-call technologies and telepresence robots have been developed, and tested among older people with and without cognitive impairments [16–18]. However telepresence robots are currently very expensive and researchers have opted to use low-cost, off-the-shelf technologies such as Skype to provide communication interventions for older people [19]. This type of socialisation intervention may be beneficial and enjoyable among older people, increasing their social networks over the long-term [19]. Skype use by adults aged 50 and over has been effective in treating depression over the long-term [20]. Similarly Mikus and Luz gave low-cost videophones to frail older residents in care homes, in order to enhance communication with their families. Although there were a number of identified technical and design problems, they demonstrated that videophones were useful and enhanced social interactions regardless of distance [16]. Boman and colleagues’ more recent study exploring the usability of videophones with older adults with dementia, revealed positive attitudes towards their use perceiving them to be worthwhile and enjoyable [18].

Retirement, living alone, living in a care environment, and cognitive ability may be associated with loneliness and isolation. These same people may also be those least likely to understand and use the technology. Although there have been some video-call intervention studies involving the elderly, many studies involve younger older adults (age 50 and above) that may not be retired, living in care, have a cognitive impairment and may have a better understanding of technology [20, 21]. This results in those who most need the intervention often being excluded from studies.

The challenge for researchers working with older people in care environments is to develop interventions that, (a) are complementary to their environment and not burdensome, (b) promote health, (c) help prevent negative health outcomes and (d) which carers can deliver. Collaborative action research (CAR) can be a useful approach for co-production of health promoting interventions with stakeholders and in particular, optimising engagement with older people, their loved ones and care staff (collaborators) to refine an intervention suited to their needs and environment [22–24]. The process of CAR typically consists of four major activities; planning, acting, observing and reflecting all derived from action research that help inform the feasibility and acceptability of an intervention, using an iterative process [25, 26]. The initial cycle of these four activities leads to a second cycle (second iteration) in which the reflections of the previous cycle (first iteration) inform the plan of the next. This can be particularly useful in identifying the barriers, facilitators and benefits of an intervention in cycle one, to further address them in cycle 2 and so forth. The CAR design allows the researchers and collaborators flexibility to go back and forth between activities, making it a useful approach in complex care environments that operate in a nonlinear system, but rather oscillate to meet the needs of their clients. As the cycles progress, a greater understanding is developed through continuous refining of methods, data collection and interpretation together with the collaborators [27]. Although there are now a number of studies using video-call interventions with loneliness and isolation as the primary outcome for older people, there is no research to date that has used CAR as an approach to implement video-calls within a care environment. Where some studies demonstrate good participant engagement with video-calls, especially for design purposes, there is a better need to understand the processes of engagement. CAR may be a useful approach to the design of a complex intervention with multiple stakeholders effecting that engagement.

**Fig. 1:**
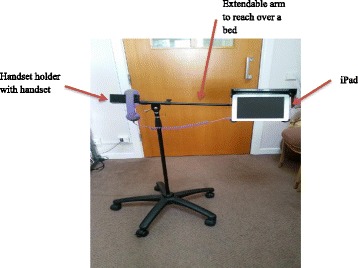
SoW device

The present study fits within the MRC framework for developing and evaluating complex interventions in that, it seeks to establish the best way to use digital communications between older people living in care environments, and their family members. The intervention ‘Skype on Wheels’ (SoW) was a simple mobile device (chassis) comprising an iPad to make video-calls using Skype, and a telephone handset (Fig. 1). If the intervention can be shown to be acceptable and feasible, then further studies can examine the effectiveness for reducing loneliness and social isolation, and improving health and wellbeing in older people. The long-term aim of this research is to explore how best to normalise [28] the use of video-calls within a care environment, through the identification of barriers and facilitators to employing video-calls with older people, staff and family to reduce loneliness and social isolation. Specifically, the study used the core activities from action research; observation on reactions and attitudes towards and use of video-calls, planning and set-up with collaborators, action of using video-calls and reflection to identify changes needed. Four objectives aligned to CAR were identified:

1. *To assess the feasibility and acceptability of using SoW among older people in care environments.* Action research allowed thorough planning of SoW implementation with collaborators to enhance feasibility and acceptability, with continuous observation of using video-calls (action) in complex environments.
2. *To identify which older people, in which care environments are able to make use of video-calls.* Observing who was able to engage in which settings after carefully planning.
3. *To identify any potential design improvements to SoW or better alternative device methods to deliver video-calls.* Observing how participants reacted to SoW current design and reflecting with collaborators on how to meet their needs.
4. *To identify the barriers, facilitators and benefits in using video-calls as perceived by staff, older people and their family contacts.* The reflective process highlighted in action research enables the identification of these.

## Methods

### Design

**Fig. 2:**
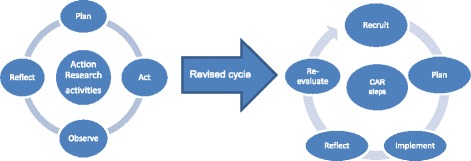
Action research cycle and revised cycle

The current study used the core activities from action research but with added activities to help better adapt to the evolving research trajectory (Fig. 2). Activities were classed as steps taken to achieve intervention implementation within a cycle: (1) *Recruitment* of older people and relevant family. This was facilitated by staff in the care environment; (2) *Planning* how best to implement the intervention. This required collaboration between the researcher, staff, older people and their family; (3) *Implementation* was the action of using video-calls. (4) *Reflection* involved feedback and identification of the barriers to and benefits of using video-calls; (5) *Re-evaluation* allowed the researcher and staff to tackle the identified barriers, and therefore inform a possible second cycle of CAR. Observing was an on-going activity that was implemented throughout the CAR steps, and so integrated within the cycle. These were employed over a 15 month study from April 2015. The cycle came to an end once all sites had entered the re-evaluation step.

### Ethics

The study was approved by the Plymouth University ethics committee in December 2013 and NHS in March 2014. All participants gave consent. Collaborators gave verbal agreement to be part of the study and notified the researchers if they did not want to provide feedback, or take part in the study. All collaborators’ information was anonymised. Participation was voluntary and participants and collaborators were assured of confidentiality.

### Recruitment of sites

The study used convenience sampling aiming to recruit care environments from Devon and Cornwall UK that had access to the internet. The concept of SoW had already been developed to some degree through student design projects led by the fourth author, and discussions with community hospital matrons and care home managers. One community hospital and six care homes continued as ‘inherited’ sites from the initial work possibly willing to participate in the current study. Additional sites were recruited using information gathered from a service improvement project carried out by the local Clinical Commissioning Group in 2014. Those care homes that had either used video-calling previously or expressed interest in using it, were contacted by email. For those who responded showing interest, an initial meeting was set up to further discuss the project. In total, eight sites were recruited over the 15 month period of the study (Table 1).

**Table 1:** Participating sites showing method, date of recruitment, care site demographics

	CH *Inherited* *April 2015*	C1 *Inherited* *April 2015*	C2 *Inherited* *April 2015*	C3 *Inherited* *May 2015*	C4 *Survey* *August 2016*	C5 *Survey* *September 2016*	C6 *Inherited* *May 2016*	C7 *Inherited* *May 2016*
No. of care staff at site	60+	45	40	30	60	15	40	40
Care staff participating	11	4	2	3	3	3	3	3
Staff turnover^a^	0%	100%	50%	100%	0%	0%	67%	100%
Education level of staff/	Degree	College	College	College	College	College	College	College
Staff wages (hourly)^b^	£10+	£8–£9	£7.50–£9	£7.50–£9	£8–£9	£7.50–£9	£8–£9	£8–£9
Average no. elderly care^c^	15	28	20	28	30	17	40	35
Minimum age of elderly	65+	65+	65+	70+	65+	70+	70+	65+
Type of care given	Acute	Dementia	Dementia	Dementia	Dementia	Dementia	Palliative	Dementia
Weekly visits^d^	Unknown	40%	25%	25%	30%	95%	30%	Unknown
No visits^e^	Unknown	15%	10%	15%	15%	1%	10%	Unknown

*CH* Community Hospital *C* care home

^a^% of recruited staff that left employment at that site during the study

^b^Against UK national minimum wage £7.30

^c^From April 2015–May 2016

^d^Estimated proportion of older people who were usually visited each week by loved one

^e^Estimated proportion of older people who usually received no visits over a 4 week period

### Participants and collaborators

Altogether, eleven NHS and 21 care home staff were collaborators (including staff turnover rates see Table 1), and 34 older people (19 residents living in a care home, and 15 patients admitted into hospital from either a care home or their own home) and 15 family members were approached about SoW. Eighteen (53%) older people (8 residents and 10 patients), and nine (60%) family members agreed to participate. Cognitive status and individual chronic conditions were not well documented during recruitment of older people; however staff preferred to include individuals without a dementia diagnosis. One resident was non-verbal and could lip read, and one resident and three patients showed early signs of cognitive decline (as reported by staff). All residents and patients were aged 65 and over and Caucasian.

### Materials

Each site was given the SoW equipment to freely use. This consisted of an iPad, a SoW device and telephone handset. Some sites had their own iPad and other sites were loaned one by the research team. A2 or A3 size posters advertising video-calls were displayed at each site, along with information leaflets for participants and staff.

### Procedures

Visits were made to each site every 3–4 weeks (on average 6 per site). Each visit represented one of the five steps in the CAR cycle. (1) *Recruitment*- staff were collaborators who helped to identify older people and family members to use Skype. (2) *Planning*- testing of equipment and WiFi connection. Staff training was provided on how to use Skype. (3) *Implementation*- staff assisted older people to use Skype with family. (4) *Reflection*- staff gave feedback using feedback sheets (after each Skype call) and face-to-face meetings with the researcher on barriers to and facilitators of the intervention. (5) *Re-evaluation*- discussion with staff on how to overcome barriers or to withdraw from the study.

Since each site varied in the way it was managed and operated, the number of times each site went through a step also varied (Table 2). Follow-up on progress and feedback from staff was also acquired by telephone or email. If a site was having difficulties during a step, an extra visit would be arranged. There was some repetition of content within the cycle, such as discussion of how best to implement the SoW device or recruitment of participants. As staff went back and forth between the steps, the intervention became more integrated into daily routines and staff became more confident in delivering it.

**Table 2:** The number of times each site was in a step during the study

	Recruitment	Planning	Implementation	Reflection	Re-evaluation	Withdrew
CH	2	2	0	2	1	Yes
C1	2	2	1	1	1	
C2	3	2	0	1	1	Yes
C3	2	1	0	1	1	Yes
C4	2	2	1	2	1	
C5	1	1	1	1	1	
C6	1	1	1	1	1	
C7	1	0	0	0	0	Yes

### Data collection

An ethnographic approach consisting of observations, unstructured interviews, memo writing, feedback forms and reflective diaries was taken towards data collection from a small number of cases. Words such as ‘alone’, ‘lonely’ and ‘isolated’ were not used during interviews with older people to avoid increasing feelings of loneliness or isolation. Unstructured interviews allowed the researcher to build rapport with the participant, rendering discussion of this sensitive topic less daunting [25]. The researcher documented all observations in note form. All conversations between collaborators and participants were anonymised, and documented into memos after each visit in a retrospective format. Additionally, with permission some conversations were documented in situ to best capture original quotes. The data were classed as field notes.

### Data analysis

Thematic analysis was used to analyse the field notes by the first researcher [26]. Saturation sampling was used, in which observations and interviews stopped when no new dominant issues were found emerging from the data. For each set of field notes, Braun and Clarke’s six phases of thematic analysis were used to gather categories which informed final themes [26]. The naming and checking of the categories, final themes and appropriate quotes were done by all of the authors. The software package NVivo version 11 was used to organise and manage the data.

## Results

### Usability

**Fig. 3:**
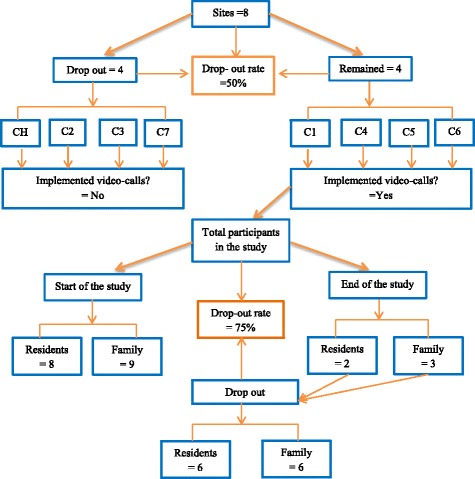
Participants and sites involved in the study

Four care homes implemented the SoW intervention and four withdrew from the study (Table 2). In total eight older people with their respective family contacts used video-calls (Fig. 3). From staff feedback, about half of the residents used video-calls once or twice a month after implementation. The remainder video-called less frequently using opportunities such as birthdays, important family occasions or when close family went on holiday. Those participants who had been using SoW but were not doing so at the end of the study had either died (*N* = 1), moved into respite care (N = 1), had their family members stop calling (*N* = 2), or did not have access to SoW due to management changes at the care home (N = 2).

### Intervention feasibility and acceptability

Observations on the feasibility and acceptability of SoW were made by the researcher or by staff, and feedback to the researcher was provided. Qualitative analysis of the field notes revealed four themes with sub-categories (Table 3). Each is discussed below with representative quotes.

**Table 3:** Identified themes and categories

Themes	Categories
1. SoW aesthetics	*1.1 Risk averseness* *1.2 Confusing technology*
2. Attitudes	2.1 *Towards technology*
	2.2 *Staff commitment*
	2.3 *Family commitment*
	2.4 *Ageism*
3. Care environment	3.1 *Patient discharge*
	3.2 *Staff turnover impact*
	3.3 *Normalisation*
4. Loneliness & isolation	4.1 *Feeling alone*
	4.2 *Capturing feelings*

### SoW aesthetics

#### Risk averseness

When the device was introduced to staff in C1, it did not appear straight forward. The activity co-ordinator was concerned about the safety of the device. Staff wheeled the device through the corridors to test its safety and were reassured that it did not pose a risk. Similarly, staff at CH refused to allow SoW on site until they were assured it had adequate safety brakes.

*“You see this bit here, it sticks out…looks sharp….I don’t know if it will be safe to wheel around the corridors… we have residents that walk up and down the narrow corridors I don’t want them to get hurt ….let’s take this around and see if it can fit through the corridors without poking anyone”.*

(Care home, activity co-ordinator)

#### Confusing technology

Patients, staff and family at CH reacted positively to the SoW device. Many of the patients who were well enough had an inquisitive approach to the device, but patients’ varying degrees of ill health affected their ability to talk with the researcher. The appearance of the SoW device caused anxiety and confusion among some residents in the care home environment. Staff reported that one resident of C1 became scared, anxious and confused as to why the device was in her room when a video-call was set up. Nonetheless, her anxiety and confusion ceased when she saw her family member on the screen, and she immediately began to make conversation. Staff suggested that the residents should ‘dress up’ the SoW device as it did not appear user friendly.

*“It looks scary and not that user friendly… maybe it should be a bit colourful with some soft material on it….put some colourful stickers and colourful wrapping around the poles”*.

(Care home, activity co-ordinator)

Unanimous feedback reported from all the care homes that implemented SoW was the non-use of the handset. The resident participating at C4 could not make use of the handset as she was hearing impaired and non-verbal; instead she used sign language to communicate. Furthermore, the activity co-ordinator at C1 explained that the sound quality was poor, creating difficulty in participating in a video-call and adding to the confusion of using a new technology. Nonetheless, staff at C1 and C4 felt the handset should remain part of the device to help residents to identify that it represents a communication service. Additionally, many patients at CH were able to identify SoW for making calls when noticing the handset and so reducing some confusion around the device. This could help those with cognitive impairments to make sense of the intervention.

Staff at C1 reported technical issues with the internet connection. On one occasion the Skype application stopped working during a video-call. Staff reported that this incident created confusion and anxiety for the resident, since she grew concerned that her family did not want to speak to her.

*“The app itself stopped working and the call got cut off… I couldn’t make a connection to call back and she became really anxious and upset….she was thinking why her family wasn’t picking up and I had to calm her down”*

(Care home, activity co-ordinator)

Staff from C6 explained residents were familiar with a larger screen and would then be more willing to participate in a video-call. Residents had a large television in their rooms that the Skype application could use. When this alternative was offered to the other care homes, all staff agreed it would be a good alternative to the SoW, additionally giving residents with visual impairments the opportunity to video-call.

*“They watch TV a lot in their rooms so they’re used to this type of screen…some have never seen an iPad before it can be a bit confusing for them”*

(Care home, manager)

### Attitudes

#### Towards technology

Staff at CH requested a ‘dummies guide to Skype’ (one A4 sheet) during a training session. Two staff members in particular felt this would be useful as they were not familiar with video-calling, and were worried they would not be able to implement the intervention. The guide was offered to all of the care homes during the planning step, but some staff felt it would not be useful. They believed that staff would not remember to use the guide, or that it would get misplaced. It was also felt that if they were to formalise the intervention by assigning detailed instructions for its use, it would become daunting for staff who would feel the need to take on yet another skill among existing duties. As well, use of the guide would reveal and possibly embarrass any staff who were under-skilled. Staff attitudes towards using technology were considered an important outcome measure for a future CAR cycle.

*“If we start telling staff they need to look at an instruction guide it’s like we’re formalising this too much…. they might get scared and worried that Oh great this is another thing I need to learn….some staff might on purpose not look at the sheet because then we’ll know they aren’t good with using technology”.*

(Care home, activity co-ordinator)

At CH, one patient decided not to Skype as she felt under-skilled in using an iPad, and concerned she would look ‘*silly*’ trying to use video-calls. Nevertheless, when it was explained she did not need any skill in using video-calls, as staff would set up the calls, she was keen to be part of the project. She still however wanted to see how other patients would use it. Older people’s lack of confidence in using technology may thus prevent participation.

*“Oh I don’t know how to use these complicated things…. I’d look silly using it …I wouldn’t bother…I think it’s a great idea so interesting but Oh not me…if I see someone else use it then I know”.*

(Community hospital, patient)

#### Staff commitment

Staff at CH explained that their busy schedules would not allow much time to implement SoW. Some care home managers also felt staff who were less confident in using SoW were less willing to commit to the project.

*“It’s hard for me ….other staff here are really busy and if they don’t really know how to use this they won’t bother much…it’s too much to have to learn while doing other things”.*

(Care home, activity co-ordinator)

Care home staff did not thoroughly engage with the feedback sheet provided. From the four care homes that began using the device, only C6 had started to complete the feedback sheet after some calls. Those staff members who used the feedback sheet said they were rushed in doing so, or would complete it later retrospectively. Staff tended to complete the feedback sheet when there was a problem related to the call. Staff reported that shorter, questions relating to specific problems about the call would be easier to complete. Due to the lack of usage, the feedback sheet data is not presented in this study as it made no significant contribution to the results.

#### Family commitment

Staff from all the homes reported difficulty in getting family to commit to video-calling. C1, C2 and C4 explained this was due to family members having busy schedules, time zone differences for contacts living abroad, along with technical issues with their own devices such as poor Wi-Fi connections abroad. In addition, staff explained residents themselves become too tired in the evening to Skype call when family members are normally available. Staff from C4 further reported that residents in turn became disinterested in the idea of using video-calls. Most significantly, many of the residents’ family members were themselves over 65 years of age, and lacked the skill to use Skype, or did not own the relevant technology. C2 found it difficult to encourage family members to join the project, therefore suspended their participation for a period, but later decided to withdraw due to the lack of family interest.

*“It’s not a matter of the residents… we just can’t get family members. With [resident] we tried to set it up but it didn’t happen …she didn’t bother to be part of it again because felt a bit let down …it’s no one’s fault though”.*

(Care home, manager)

#### Ageism

One family member at CH highlighted the issue of ageism evidencing the belief that older people cannot make use of technology. The family member explained that due to her mother’s age (90+) she would not be able to use any technology, that she would not want to stay in touch with her other family members, and that she herself visits her regularly. In addition, as the care home staff were ultimately responsible for authorising recruitment of participants to the project, a number of residents were not approached and consequently missed the opportunity to join the study. A common justification was that those residents with dementia will not be able to cope with new technology.

*“I don’t want to involve* [residents] *because of their cognitive impairment they won’t be able to understand what’s going on…I’m not sure how they will react so it’s best to not”.*

(Care home, activity co-ordinator)

Similarly, in some of the care homes, those who had hearing, visual impairments, or were non-verbal were not approached about the study by staff. Nonetheless, C4 had successfully recruited one resident who was non-verbal. This resident was able to communicate with family using sign language. Staff explained that the resident now had a way to stay in touch with distant relatives who previously wrote letters or sent text messages, whereas now the resident was able to see her relatives and their surroundings in real time, something a telephone call or text message was not able to achieve.

*“She has family who moved to* [abroad] *recently…they always try to describe how lovely their home is…they write to her…now she can actually see what it all looks like and it was great…she holds up her things to the screen… really loves it…yeah they* [family] *all use sign language …no issues so far”.*

(Care home, manager)

### Care environment

#### Patient discharge

In the CH setting, patient hospitalisation would normally last no more than a couple of weeks, and most would be discharged after one week. Most patients would have left the facility by the time the device was presented to them, family members were contacted, and then set-up to use video-calls. It is evident that an intervention such as this is difficult to implement in a short term care-setting. Hospitals may require an alternative method of implementation in comparison to a long term care-setting.

#### Staff turnover impact

Four care homes had changes in management and site staff. This in turn slowed down the progress of the study due to having periods of no communication between the researcher and the site, or not being able to visit until the site was back to its ‘normal’ running. This resulted in some sites having to revert to the recruitment step when new staff were appointed. With these changes, some valuable information was lost such as Skype log in details, feedback sheets or recruitment posters. Most importantly, however, residents who had been using Skype were no longer able to.

#### Normalisation

C1 and C6 provided a busy, activity focused environment for their residents. Both had daily scheduled activities where SoW became part of those scheduled activities, and was integrated on to their activities board and into weekly newsletters. Staff at these homes felt it would be easier to normalise the intervention if it was seen as just another on-going activity that they provided.

*“I think we will put this up on the activity board with the rest… that way it will just be another normal thing…if it’s in the newsletter then the families will also see this”.*

(Care home, activity co-ordinator)

### Loneliness and social isolation

#### Feeling alone

Although trigger words such as ‘alone’, ‘lonely’ and ‘isolated’ were avoided during conversations with older people, feelings of being lonely and isolated were made apparent. Three patients at CH expressed feelings of loneliness during interviews with the researcher. One patient explained she felt bored due to lack of interaction. She became upset that she was in a hospital environment, and her situation reminded her that her family were far away. She became tearful, but was hopeful that the SoW device could help her to reconnect with some of her distant family as she felt alone in the hospital.

*“I do get bored… I don’t have anyone to talk to…I have family that visit once in a while…I’m here now…I’m not well and I feel alone…I have family I would like to see…Yes I think it’s a great idea this”.*

(Community hospital, patient)

The second patient explained that she often sees her children, but would like to have the chance to see her infant great grandchild. She became slightly upset that she still had not seen her great grandchild, and felt left out by her family. She was excited at the thought of being set-up on SoW where she could finally see her family.

*“Oh yes… my daughters come to see me even here at the hospital…but I haven’t had the chance to see the little one yet…that’s my granddaughter’s little one… they live too far away…I wish I had the chance to see”.*

(Community hospital, patient)

The third patient overheard some of the conversations between the researcher and patients, and was keen to get set-up on the SoW to reconnect with her family. In contrast, of the patients who did not want to use SoW, one explained that she did not want her family members to see her looking unwell even though she misses them. She was worried that they would become upset by her current appearance. Although feelings of loneliness may reduce for some people, families may become distressed as they watch their loved one’s health deteriorate.

#### Capturing feelings

When speaking with older people about the possibilities of reconnecting with family and friends, feelings of loneliness and isolation were evident and captured in field notes. The feedback sheet after each call acted as a source of documenting any changes in mood such as feeling happier and less isolated. However, as previously mentioned, staff members did not record this information during the study. It was only identified that some older people were feeling lonely and isolated through conversations with the researcher, or by staff identifying them as being lonely individuals who might be a good candidate for SoW. Staff from C1 suggested that in order to best capture these feelings, simplified scales ought to be developed, as residents have previously enjoyed completing questionnaires, and it would be an easier way to document any changes. For future iterations of this study, loneliness and isolation will be considered as key outcome measures. In addition, some residents may have been unwell and therefore an important outcome measure of well-being would be advantageous to include.

##### Barriers towards implementation

Key aspects of the results highlight the lack of sustained use of SoW across sites for various reasons. Five key barriers towards implementing the intervention were identified (Table 4).

**Table 4:** Barriers and suggested next steps

Barriers	Suggested next steps (Re-evaluation)
(1) Staff turnover	High staff turnover meant lack of sustained use of SoW. There is a need to engage more staff at each site.
(2) Risk averseness	Perceptions of the device being unsafe and risky to use in a care environment were noted. There is a need to conduct a risk assessment on site to demonstrate the safety of the device before use. In addition staff training to reduce perceptions of risk that override implementation.
(3) Intervention design	The SoW device did not appear user-friendly to some residents, therefore staff suggested there is a need to redesign it. Staff wanted to provide video-calls on a larger screen such as a TV because residents are more familiar with it, compared to an iPad.
(4) Family commitment	Staff reported that some relatives stopped video-calling because they may have been unsure of what to talk about, therefore a conversation aid is needed. C1, C4, C6 felt there should be additional social contacts other than family to video-call with to increase their social networks and reduce loneliness.
(5) Staff attitudes towards intervention implementation	Not all staff members committed to the project. Some staff felt they needed more training in how to use the intervention. Staff leading the project felt there is a need to target those who are not confident in using technology without causing embarrassment. Also, adherence to completing the feedback sheet by staff was low because it was not made a priority.

## Discussion

This study addressed four objectives. It found that older people and their family contacts are capable of using SoW and found it beneficial however, the feasibility of its use by those with cognitive impairments is yet to be determined. A long-term care environment may be more suitable for the on-going use of video-calls by older people, compared to hospital settings. However, older people in the hospital environment felt video-calls could be useful to them, suggesting maybe an alternative approach in implementation that meets the needs of a hospital environment. There is a need to re-design the SoW device and provide video-calls on a larger screen as an alternative, and reduce perceptions of risk towards the device. Staff reflection identified five key barriers towards the lack of sustained use of video-calls that need to be addressed through further cycles of action.

Overall the finding that older people are happy and keen to use video-call technology is consistent with previous research [16–18, 27, 29]. Relative to other forms of technology to reduce loneliness for residents such as telepresence robots [30, 31], video-calls are inexpensive. Telepresence technologies can cost thousands of pounds which do not reflect the need for cost effective interventions [32]. The current intervention has the potential for application in a variety of care environments allowing its routine use. An ethnographic approach employed over a long-term period across a number of sites gathered a large, rich dataset through continued observation, reflection and interviews. Key findings related to lack of sustained and routine use across sites which resulted from staff engagement and turnover, risk averseness, family attitudes, the SoW design and loneliness which are discussed sequentially.

Foremost, the current study had problems with usability of SoW and retaining sites throughout the cycle. The most significant and relevant finding from the field note data was the staff turnover rates and site drop-outs. Most care homes were under-staffed with some moving between sites to help manage the workload and a high turn-over. Lack of skills, self-efficacy and negative attitudes towards technology may not be the only contributors as to why staff were not committed to the project. Staff appeared so short of time that they could not commit to the project regardless of their attitudes and therefore was a significant finding explaining the lack of sustained use across sites. Implementing interventions can become an onerous task and burdensome for those care homes that are under staffed, explaining why only two residents on average per site were using SoW and some were unable to continue its use. Evidently, video-calls were a lower priority for busy staff who were focussing on primary care aspects until their care home was normalised (enough staff working on site). The non-use of SoW at sites that had dropped out reflects the social and organisational factors associated with care environments and intervention implementation. Other than staff turnover, some researchers believe that stakeholders lack agreement of what the ‘organising vision’ of ‘ageing in place’ is for health services alike and so impacts implementation of such interventions [33]. Even so, where stakeholders are successful in agreeing to that vision, implementation can be compromised if important barriers are not over-come [34], in this case the high staff turnover and low engagement. Specifically, Greenhalgh and colleagues emphasise that if the needs of older people are not adequately met, then care providers should increase resources to support those needs from an organisation standpoint, rather than researcher led [34]. Sites where SoW was better accepted by staff embodied an activity led environment and staff were accustomed to dedicating time to engage with activities, thus becoming a normal part of their care duties. It appears that normalisation of an intervention can only occur within a normalised care environment.

Another contributing barrier towards implementation of SoW was the perception of risk it posed. Albeit the nature of care staff working with vulnerable individuals is to minimise risk however, a risk aversive stance towards adopting a new potentially useful intervention may override the risk in reducing loneliness. This finding is not uncommon particularly among technological interventions in UK health settings where the social construction of risk can minimise or halt implementation into practice [35]. In the current study staff (social actors) adopted a technical approach towards risk assessment where the risk was placed within the device itself. That is, risks were found in the design and so it was important to ‘test’ SoW’s safety to reduce physical harm [36]. Alternatively, some staff adopted a systematic approach towards risk assessment where during the implementation of SoW, risk emerged from the level of technology acceptance, resources available and management of conflicting interests in sustaining it [35, 36]. This further explains the lack of staff engagement and why some sites withdrew. Taylor and colleagues’ suggest that further research is needed to explore if training can impact on the professional practice of those with less favourable beliefs about the intervention [22], or need to explore the predetermined roles and values of care staff towards technology acceptance. Therefore, capturing staff attitudes towards video-calls before implementation is recommended.

### Limitations of the study

The finding that family members were unable to commit to video-calls is a major drawback to an intervention intended to reconnect families. To date there has been no research that examines how the lack of family commitment to stay connected with residents in long-term care, can affect key outcomes such as loneliness and social isolation. Gaugler’s findings from a synthesis and critical review on family involvement in long-term care, urged that future research should recognise and include residents without family support, and how external social contacts can influence key outcomes of the study [37]. Befriending interventions with older people have proved valuable in increasing social networks and reducing social isolation [4, 5]. The concept of including external social contacts in further CAR cycles has been identified within the findings of the current study.

The design of SoW was not yet optimal for the residents’ needs as some found it was an intimidating or even frightening piece of technology. This highlights the importance of the ‘materiality of technology’ where material features of devices such as the shape, colour and overall likeability can have a powerful influence on the usability and acceptability of a new intervention [38]. The likeability of the device is important as the way video-call technology is delivered to a generation who are not very confident in using it, will directly affect the number of older people who decide to participate. Older people may benefit from using video-calls but could reject the opportunity due to the poor design of the intervention. The design needs have been well documented and the device can be re-designed using focus groups. The use of focus groups to evaluate internet interventions [39] and video-call technology with older adults has proved advantageous for other researchers [40]. Moreover, a surprising finding about SoW was that although the handset was not used during calls, it still helped to identify that SoW was a tele-service. For an older generation, recognisable props can help make sense of the intervention. Similarly, the idea of providing Skype through familiar technology such as TV may increase the usability of video-calls among older people. Referring back to the ‘materiality’ view of interventions, there are sociological implications inferred from iPad use. That is, they can have cultural meanings where a relatively newer technology that uses iPad’s can symbolise modernity, status and youth especially to an older unexperienced generation [38]. Others, such as telephone handsets and TV’s may represent familiarity and simplicity.

Although terms such as ‘lonely, ‘alone’, or ‘isolated’ were avoided when speaking with older people, some were still reminded of their situation which undoubtedly caused some distress. This indicated that individuals may have in fact been feeling lonely and isolated. Furthermore, video-calls could in turn increase supplementary negative emotions for families that will see their loved ones in possible ill health. For that reason hospital settings where older people are at their most vulnerable in ill health, may not be a suitable environment to employ video-calls.

Other notable findings were that staff recruited residents who had better mental health, were less likely to have cognitive impairments, would be more responsive and willing to use video-calls, and with low levels of physical and sensory impairments. Also, residents with dementia may have been excluded. Care home staff emphasised the importance of issues concerning capacity and consent for their residents and wanted to first validate the feasibility and acceptability of the intervention among those with no noticeable cognitive decline. Other researchers have found that those with cognitive impairments do not benefit from being involved in the early developmental stages of an intervention which could have a negative impact. That is, poorly functioning technology can cause obfuscation and even frustration for elderly people [32].

Additionally, the mental and physical impairments of older people were not documented well by staff. For many older people, changes in mental and physical impairments can be common, thus having an impact on their ability to use video-calls. Therefore there is a need to prioritise and emphasise the importance of accurately documenting this information. Even so, the current study revealed that some older people with physical impairments such as being non-verbal can still use video-calls, allowing a more useful method of communication.

It is important to note that due to the target participant group and study environment, high drop-out rates and small sample sizes are common for such studies. In addition, all participants resided in Devon and Cornwall which are demographically largely white Caucasian, not allowing for any ethnic diversity within the sample. Although the sample was small, the data collected in the study was considered sufficient to cover the study aims and objectives and provide a rich, in-depth account of experiences. Nonetheless, generalisations of the findings should be carefully made.

Unequivocally, the type of culture and environment each care home has, such as the type of residents and their contacts, staff attitudes and resources and the intervention itself can affect the success of implementation. This study highlighted the complex reality of implementing technological interventions into practice where many of the barriers reflected the social environment and organisation in which participants resided. It is known that many interventions will not reach its target population or the target population may not adopt it as they are ‘imposed from the outside’ due to the ‘limited organisational support’ or ‘organisational instability’ [41]. Consequently, there was a need to study important participant characteristic of staff skills, working conditions, quality of family networks and readiness of technology acceptance and organisational change to help improve intervention implementation.

## Conclusion

Institutional and older peoples’ participation was low due to high staff turnover, implementation was not possible in four out of the eight study settings which had accepted to participate, there was considerable lack of engagement of families and lack of motivation of the care homes’ staff to complete the study procedures. However, for those older people who used video-calls they appeared very beneficial. The findings from this CAR study support the need for further exploration of video-calls for older people with and without cognitive impairments in care homes, to optimise engagement, before any rigorous evaluation of the effectiveness of SoW to reduce loneliness and social isolation.

## Abbreviations

CAR: Collaborative action research
SoW: Skype on Wheels
